# Association between cytokines and suicidality in patients with psychosis: A multicentre longitudinal analysis

**DOI:** 10.1016/j.bbih.2024.100756

**Published:** 2024-03-20

**Authors:** Gunnhild E. Hoprekstad, Silje Skrede, Christoffer Bartz-Johannessen, Inge Joa, Solveig K. Reitan, Vidar M. Steen, Anja Torsvik, Erik Johnsen, Rune A. Kroken, Maria Rettenbacher

**Affiliations:** a Division of Psychiatry, Haukeland University Hospital, Bergen, Norway; b Department of Clinical Medicine, University of Bergen, Bergen, Norway; c Norwegian Centre for Mental Disorders Research (NORMENT), Haukeland University Hospital, Bergen, Norway; d Section of Clinical Pharmacology, Department of Medical Biochemistry and Pharmacology, Haukeland University Hospital, Bergen, Norway; e TIPS, Network for Clinical Research in Psychosis, Stavanger University Hospital, Stavanger, Norway; f Department of Public Health, Faculty of Health Science, University of Stavanger, Stavanger, Norway; g St. Olav's University Hospital, Department of Mental Health, Nidelv DPS, Trondheim, Norway; h Norwegian University of Science and Technology, Department of Mental Health, Trondheim, Norway; i Department of Clinical Science, University of Bergen, 5020, Bergen, Norway; j Dr. Einar Martens Research Group for Biological Psychiatry, Department of Medical Genetics, Haukeland University Hospital, Bergen, Norway; k Medizinische Universität Innsbruck, Austria

**Keywords:** Cytokine, Depression, Inflammation, Interleukin, Psychosis, Schizophrenia, Suicidality, T-cells

## Abstract

Suicide is a common cause of death in all phases of schizophrenia spectrum disorder, particularly in the youngest patients. Clinical measures have demonstrated limited value in suicide prediction, spurring the search for potential biomarkers. The causes of suicidal behaviour are complex, but the immune system seems to be involved as it reflects or even causes mental suffering. We aimed to identify cytokines with associations to suicidality in a sample of patients with symptoms of active psychosis. Patients with schizophrenia spectrum disorder (N = 144) participating in a semi-randomized antipsychotic drug trial (the BeSt InTro study) were assessed with the Positive and Negative Syndrome Scale (PANSS) and the Calgary Depression Scale for Schizophrenia (CDSS) at eight visits across 12 months. The Clinical Global Impression for Severity of Suicidality scale (CGI-SS) was used for assessing suicidality. Serum concentrations of tumour necrosis factor (TNF)-alpha, interferon (IFN)-gamma, interleukin (IL)-1beta, IL-2, IL-4, IL-6, and IL-10 were measured using immunoassays. A logistic regression model was used to investigate the association between cytokine levels and suicidality. To enhance clinical significance, the CGI-SS scores were dichotomized into two groups before analyses: low (=1) and high (≥2) risk for suicidality. Both uni- and multi-variate analyses revealed an inverse correlation between IL-2 and IL-10 serum levels and suicidality, where lower cytokine concentrations of IL-2 and IL-10 were associated with higher suicidality scores. The results were consistent when adjusted for depression and substance use. These results indicate that inflammatory processes are linked to the risk of suicidality in patients with schizophrenia spectrum disorders.

## Highlights

• Suicidality is a major challenge in schizophrenia.
• Biomarkers could aid prediction of suicidality.
• We investigated serum cytokine levels and suicidality in schizophrenia.
• During a 1-year follow-up, IL-2 and IL-10 were inversely correlated to suicidality.

## Abbreviations

AP –: antipsychotic medication
BMI –: Body mass index
CDSS –: the Calgary Depression Scale for Schizophrenia
CNS –: the Central nervous system
CGI-SS –: the Clinical Global Impression for Severity of Suicidality scale
CRP –: C-reactive protein
GWAS –: Genome-wide association study
ICD-10 –: the International Classification of Diseases 10th Revision
ICH GCP –: the International Conference on Harmonisation (ICH) of Good Clinical Practice (GCP) standards
IFN –: Interferon
IL –: Interleukin
NK cells –: Natural killer cells
PANSS –: the Positive and Negative Syndrome Scale
pg/mL –: picograms per millilitre
SCID-1 –: the Structured Clinical Interview for DSM-IV Axis 1 Disorders
SCI-PANSS –: the Structured Clinical Interview for the PANSS
SD –: Standard deviation
SUD –: Substance use disorder
Treg –: regulatory T-lymphocyte
TNF –: Tumour necrosis factor
WHO –: World Health Organization

## 1 Introduction

Suicidality is one of the most demanding topics in psychiatry. Globally, over 800,000 people commit suicide every year (World Health Organization, 2014), and suicide was recently listed as the main cause of premature death for adolescents suffering from psychosis (Barbeito et al., 2021). Risk factors associated with suicide are complex and include the psychotic symptoms themselves (Stefenson and Titelman, 2016; Hawton et al., 2005), substance and alcohol use (Karnick et al., 2021; Kamali et al., 2000), the male sex (Franklin et al., 2017), and socio-economic issues (Song and Lee, 2016). Comorbid depression may contribute to suicidality (Upthegrove et al., 2017; Hawton et al., 2013). Suicidal ideation can also be present independently of depression in patients with psychotic symptoms (Mellesdal et al., 2010), manifesting as hallucinations, and unspecific symptoms like agitation and anxiety can contribute (Stanley et al., 2018; Black and Miller, 2015; Kjelby et al., 2015; Harkavy et al., 2003). The prediction of suicidal behaviour based on clinical symptoms has proven insufficient in the general population (Nock et al., 2022; Large, 2018), and even more so in psychosis (McGirr and Turecki, 2008; Fredriksen et al., 2020; Docherty et al., 2022). Accordingly, there is an urgent need to identify other predictors of suicidal behaviour to guide proper interventions. Furthermore, the exploration of biological factors improves the understanding of mental suffering.

Associations between psychosis and inflammation are well established (Raison et al., 2006; Muller, 2018; Rengasamy et al., 2020; Haroon et al., 2020; Sha et al., 2022). The underlying pathological mechanisms are only partially understood, although messenger molecules from the immune system such as cytokines seem to be involved (Upthegrove and Khandaker, 2020). Peripheral cytokines are expressed in low, homeostatic concentrations, and in the case of immune challenges, both cytokines and immune cells can cross the blood-brain-barrier and enter the central nervous system (CNS) (Daneman and Prat, 2015). Additionally, cytokines can be locally produced in the CNS by neurons and microglia (Ransohoff and Benveniste, 2019). Cytokines contribute to the intercellular communication, development and activation of lymphocytes, and they mediate several inflammatory processes and apoptosis (Kuypers, 2022). They may be divided into pro-inflammatory (e.g., interleukin [IL]-1, IL-2, IL-6, tumour necrosis factor alpha [TNF-α], interferon gamma [IFN-γ]) and anti-inflammatory cytokines (e.g., IL-4, IL-10), although their functions partly overlap (Ransohoff and Benveniste, 2019) and the interplay is complex (Cavaillon, 2001): IL-2 was originally identified as a pro-inflammatory cytokine but is now recognized also as a pleiotropic cytokine, possessing a wider range of differentiation and modulating abilities than first anticipated (Liao et al., 2011). Since the neurons themselves express receptors for cytokines, cytokines may also play a potential role in neuronal transmission and affect the metabolism of neurotransmitters (Kettenmann and Verkhratsky, 2011; Kettenmann et al., 2011).

Inflammation and cytokine variations in schizophrenia spectrum disorder are documented (Muller, 2018; Hoprekstad et al., 2023; Dawidowski et al., 2021), and such alterations have been reported both with and without comorbid depression (Goldstein et al., 2015; Noto et al., 2011; Khandaker et al., 2017). Inflammatory processes could possibly trigger depression and destructive emotions, contributing to suicidality (Huang et al., 2022; Jiang et al., 2022). However, independent of depression, cytokines have been postulated to exert direct effects on dopaminergic metabolism in the brain and indirect effects on glutamatergic neurotransmission through tryptophan catabolism (Miller et al., 2011; Munkholm et al., 2013; Marcinowicz et al., 2021; Kristiansen et al., 2007). Recent research has revealed biological factors associated with severe suffering, leading to an ongoing search for biomarkers that could predict severe suicidality (Christodoulou et al., 2012; Li et al., 2023) and link suicidality to aberrant cytokine levels (Black and Miller, 2015; Gananca et al., 2016). Increased levels of IL-1β (Black and Miller, 2015), IL-6, and TNF-α have been associated with suicidal behaviour (Janelidze et al., 2011), as has a decreased level of IL-2 (Gananca et al., 2016; Janelidze et al., 2011). Furthermore, one study found an association between IL-6 and suicide attempts with high impulsivity and sensation-seeking personality traits (Isung et al., 2014; Siegel et al., 1999).

Some studies have examined the relationship between cytokine activity and aggression or impulsivity, often linked to suicidal behaviour, in depression (Coryell et al., 2018). The association between aggression, anger, hostility, and cytokines, independent of depressive symptoms, was also shown in a patient sample receiving cytokine-based immunotherapy (Hasanov et al., 2021). Again, cytokines are postulated to play a role in suicidal behaviour, beyond their role in promoting depressive symptoms (Coryell et al., 2018; Ducasse et al., 2015; Serafini et al., 2013). As concluded in a recent network meta-analysis, mediators of stress could also be of relevance when searching for neurobiological correlates of suicidality, especially in genetically vulnerable individuals (Thomas et al., 2021).

A potentially confounding factor in patients with psychotic disorder is substance use, both in the context of suicidality and in the context of cytokines. Recent findings point towards a correlation between substance use disorders (SUDs) and cytokines; the associations are however inconsistent for individual cytokines (Coller and Hutchinson, 2012; Maza-Quiroga et al., 2017; Zahr, 2018; Kuo et al., 2018), and further investigations are needed. In animal models, both ethanol (Qin and Crews, 2012) and morphine administration led to enhanced IL-6 and IL-1β levels (Raghavendra et al., 2004). In humans, both cocaine (Moreira et al., 2016) and cannabis use is associated with elevated IL-6 (Ajrawat et al., 2024). Finally, antipsychotic drugs may differentially impact levels of inflammatory markers (Fathian et al., 2022).

Overall, the existing evidence is equivocal regarding cytokines as candidate biomarkers for suicidal behaviour in psychosis. The aim of this study was therefore to longitudinally examine the association between suicidality and cytokines in a large patient cohort followed during 1 year of treatment for active-phase psychosis and with thorough clinical characterization combined with longitudinal measurements of serum cytokines using a validated, high-sensitivity assay.

## 2 Methods

### 2.1 Study design

The studied cohort was part of the Bergen-Stavanger-Innsbruck-Trondheim (BeSt InTro): a naturalistic, semi-randomized, rater-blinded comparison between the three atypical antipsychotic drugs olanzapine, aripiprazole, and amisulpride (Johnsen et al., 2020). Three study sites in Norway and one site in Austria recruited the participants. Inclusion took place between 20 October 2011 and 30 December 2016. Data collection was completed 21 December 2017.

### 2.2 Ethics

The ethical principles of the Declaration of Helsinki were applied in all phases of the study. The study was approved by the Regional Ethical Committees for Medical and Health Research Ethics in Norway and the Norwegian Medicines Agency, and likewise approved by the Etikkommission der Medizinische Universität Innsbruck and the Austrian Federal Office for Safety in Health Care (BASG) in Austria. After the attending psychiatrist or physician confirmed that the patients had the capacity to provide informed consent, all participants gave their written informed consent prior to study inclusion. Clinical monitoring was in line with the International Conference on Harmonisation of Good Clinical Practice (ICH GCP) standards, supplied by the Department of Research and Development, Haukeland University Hospital in Norway, and by the Clinical Trial Centre at the Medical University of Innsbruck, Austria.

### 2.3 Participants and inclusion and exclusion criteria

Inclusion criteria for the BeSt InTro study were that participants were at least 18 years of age, able to cooperate with oral antipsychotic treatment, and had a diagnosis within the F20-F29 chapter in the International Statistical Classification of Diseases 10th Revision (ICD-10) (WHO, 1994). Furthermore, the participants had a score of 4 or more on at least one of the following items of the Positive and Negative Syndrome Scale (PANSS) (Kay et al., 1987): P1/delusions, P3/hallucinations, P5/grandiosity, P6/suspiciousness/persecution, or G9/unusual thought content, indicating an active phase of psychosis. These PANSS items evaluate core schizophrenia symptoms (Lehoux et al., 2009). All personnel assessing patients in the study were trained and certified via the PANSS Institute (New York, USA) in order to secure high inter-rater reliability. Exclusion criteria were pregnancy or breast feeding, inability to understand the native language, organic psychosis, hypersensitivity to the active substances in the study drugs, or somatic contraindications. Both in- and outpatients could participate in the study.

### 2.4 Clinical variables and outcomes

Study participants were assessed at eight study visits over a 12-month period, including at baseline followed by weeks 1, 3, 6, 12, 26, 39, and 52. Medical history, smoking habits, alcohol and drug use, as well as height, weight, and blood pressure were recorded. The levels of psychotic symptoms were assessed using the PANSS. The Clinical Global Impression for Severity of Suicidality (CGI-SS) psychometric rating scale was used for measurements of suicidality (Lindenmayer et al., 2003; Busner and Targum, 2007). This is a semi-structured interview for clinicians investigating actual, recent, and past suicidality. Result categories are not at all suicidal (1), mildly suicidal (2), moderately suicidal (3), severely suicidal (4), and attempted suicide (5), measuring the most severe suicidality experienced in the prior 7 days. At each visit, study participants were classified either as “non-suicidal” when given a CGI-SS score of 1, or “suicidal” when given a CGI-SS score of 2 to 5. Depression was measured using the Calgary Depression Scale (CDSS) (Addington et al., 1996). A CDSS score above 6 predicts the presence of a major depressive episode with high specificity and sensitivity (Addington et al., 1992).

### 2.5 Blood sample collection and analysis of cytokines

Fasting-state peripheral blood was collected between 8 and 10 a.m. at all eight study visits. Samples were kept at room temperature for 20-120 min, followed by centrifugation at 3300 rpm for 10 min. The serum was stored at -80 °C until analysis. Samples were thawed on ice. IL-1β, IL-2, IL-4, IL-6, IL-10, IFN-γ, and TNF-α were analysed via multiplex immunoassay using the High Sensitivity 9-Plex Human ProcartaPlex™ Panel (ThermoFisher Scientific, Waltham, MA, USA). The assay was prepared according to the manufacturer's protocol, except from the replacement of the universal assay buffer with PBS/0.1% Tween to reduce matrix effects. Serum samples were randomized across plates with respect to the antipsychotic medication use and participants' sex, but with all samples from each study participant (visits 1-8) on the same plate. Data were acquired with a Luminex 200 instrument (Luminex, Austin, TX, USA). To quantify analyte concentration and to calibrate between-plate variability, a standard curve was included on each plate. In addition, we included a reference sample of pooled serum from five healthy controls on all runs to control for batch effects. All samples were run in duplicate, and the results were given as pg/mL. Cytokine analyses from the same sample are previously described (Hoprekstad et al., 2023).

### 2.6 Statistical analysis

The data in this study were collected longitudinally, and the statistical software R was used for the analyses (R Core team, 2023). In the analyses, all observations were used, hence patients who completed the study contributed during eight observations. CGI-SS and cytokine-values were measured at each time point and could vary within the patient during the follow-up period. For each observation, a patient's CGI-SS result was dichotomized into low (=1) or high (≥2) risk for suicidality. Dichotomization was done to simplify clinical interpretation and relevance, comparing cytokine levels in patient-observations with low versus high risk of suicidality.

A logistic regression model fitted to all the observations was used to investigate the effects of the different cytokines on suicidality. The following six cytokines were investigated: IL-2, IL-4, IL-6, IL-10, IFN-γ, and TNF-α. Due to high skewness in the data, the cytokine values were log-transformed. Some of the cytokines were highly correlated, as expected. Therefore, we created separate models for each cytokine, that is models where one cytokine at a time was included as an explanatory variable (univariate analyses). In addition, a multivariate model including all the six cytokines simultaneously was analysed to examine whether the effect of each individual cytokine changed when adjusted for the others. Age, sex, state of depression (per the CDSS), and randomized antipsychotic medication were included as covariates in all models. A random intercept for each individual was added in all models to account for dependencies in the data due to repeated measurements from study participants.

We performed a sensitivity analysis where we also included C-reactive protein (CRP), Body mass index (BMI), and substance use in the model. Smoking is a known confounder in the context of severe mental disorders and immune marker analyses; hence we performed a sensitivity analysis where smoking was included as a covariate in the statistical model. A sensitivity analysis using only the baseline observations was also performed.

Furthermore, we performed a sensitivity analysis where we replaced the outcome variable based on CGI-SS scores with an outcome variable based on the CDSS item 8, suicidality, to test if the relationship between cytokines and suicidality remained the same when another clinically relevant measure for suicidality was used. CDSS item 8 results were also dichotomized into low (0) and high (≥1) risk of suicidality.

Finally, we compared the cytokine levels in those with and without substance use or dependence.

## 3 Results

A total of 144 patients were included in the BeSt InTro study. Due to missing data for three participants, further analyses included 141 patients (see Table 1). The majority was diagnosed with F20/schizophrenia (58%) or F22/delusional disorder (15%). The mean PANSS total score with standard deviation (SD) at baseline was 75.9 (15.0), and the mean CDSS score was 6.5 (5.1). Baseline measures for the two groups were compared. We found a significant statistical difference between CDSS, PANSS total, and PANSS general scores (see Table 1). One patient committed suicide during the follow-up period (Johnsen et al., 2020).

**Table 1:** Demographic information and relevant measures at baseline (N = 141).

	All (N = 141)	Non-suicidal (N = 89, 37%)	Suicidal (N = 52, 63%)	P-values
	Number/Total number (percentage)			
Women	49/141 (35%)	27/89 (30%)	22/52 (42%)	0.199
Men	92/141 (65%)	62/89 (70%)	30/52 (58%)	0.199
F20: Schizophrenia	81/141 (57%)	49/89 (55%)	32/52 (62%)	0.484
F21: Schizotypal disorder	2/141 (1%)	1/89 (1%)	1/52 (2%)	1
F22: Persistent delusional disorders	21/141 (15%)	15/89 (17%)	6/52 (12%)	0.218
F23: Acute and transient psychotic disorders	18/141 (13%)	12/89 (13%)	6/52 (12%)	0.26
F25: Schizoaffective disorders	10/141 (7%)	6/89 (7%)	4/52 (8%)	0.373
F28: Other non-organic psychoses	1/141 (1%)	0/89 (0%)	1/52 (2%)	1
F29: Unspecified non-organic psychosis	8/141 (6%)	6/89 (7%)	2/52 (4%)	0.373
Immune diseases^a^	8/141 (6%)	4/89 (4%)	4/52 (8%)	0.467
Smoking	72/141 (51%)	41/89 (46%)	31/52 (60%)	0.067
Alcohol non-use/without function loss	123/141 (87%)	77/89 (87%)	46/52 (88%)	0.8
Alcohol misuse or dependence	14/141 (10%)	9/89 (10%)	5/52 (10%)	1
Drug non-use/without function loss	108/141 (77%)	67/89 (75%)	41/52 (79%)	0.684
Drug misuse or dependence^b^	29/141 (21%)	19/89 (21%)	10/52 (19%)	0.832
Cannabis	46/141 (33%)	29/89 (33%)	17/52 (33%)	1
Antipsychotic naivety at inclusion	56/141 (40%)	35/89 (39%)	21/52 (40%)	1
	Mean (SD)			
Age	31.9 (12.8)	32.6 (13.2)	30.5 (12.1)	0.332
Age at psychosis onset	24.7 (9.1)	25.2 (9.1)	24 (9.1)	0.5
PANSS total score	75.9 (15)	72.9 (16.2)	81.3 (11)	<0.001
PANSS positive score	20.6 (4.5)	20.3 (4.9)	21.2 (3.9)	0.247
PANSS negative score	17.1 (5.7)	16.8 (6)	17.7 (5.2)	0.326
PANSS general score	38.2 (8.4)	35.8 (8.4)	42.4 (6.7)	<0.001
CDSS score (state of depression)	6.5 (5.1)	4.4 (4.3)	10.2 (4.4)	<0.001
BMI (kg/m^2^)	25.4 (5.9)	24.9 (6.1)	26.2 (5.6)	0.256
CRP (mg/L)	2.2 (4)	1.7 (2.8)	3.1 (5.5)	0.108

Note.

a Immune diseases (n): coeliac disease (1), Crohn's disease (1), psoriasis (3), unknown autoimmune disease (3).

b Drug misuse/dependence, mainly cannabis use (Skrede, 2021). Body mass index (BMI). Calgary Depression Scale for Schizophrenia (CDSS). C-reactive protein (CRP). Positive and Negative Syndrome Scale (PANSS).

**Fig. 1:**
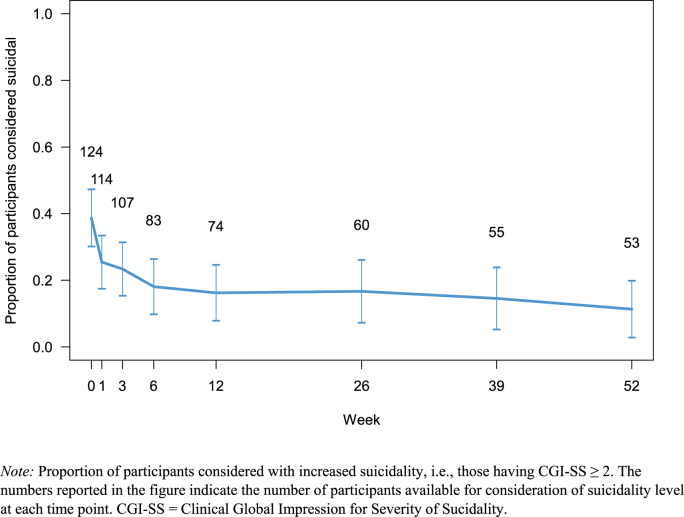
Proportion of participants considered with increased suicidality (CGI-SS ≥2). *Note:* Proportion of participants considered with increased suicidality, i.e., those having CGI-SS ≥ 2. The numbers reported in the figure indicate the number of participants available for consideration of suicidality level at each time point. CGI-SS = Clinical Global Impression for Severity of Sucidality.

**Fig. 2:**
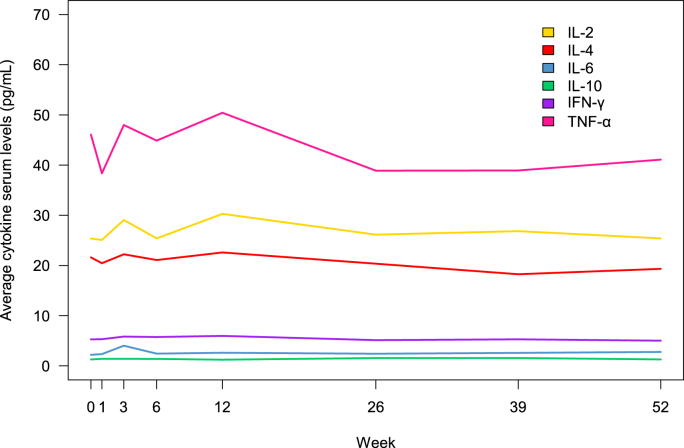
Average serum levels of cytokines at 1-year follow-up.

A total of 670 measures of suicidality were made during the year of follow-up, of which 153 observations indicated elevated suicidality (patients exhibiting a CGI-SS score ≥2). During the first 6 weeks of observation, the mean suicidality score at a group level dropped, while it remained stable for the remaining 48 week-follow-up period (see Fig. 1). At a group level, serum levels of IFN-γ and IL-10 were relatively stable throughout the follow-up period of 52 weeks (see Fig. 2). For IL-2, IL-4, IL-6, and TNF-α, fluctuations were observed between time points, particularly 0 and 12 weeks, followed by a phase of stable levels.

**Fig. 3:**
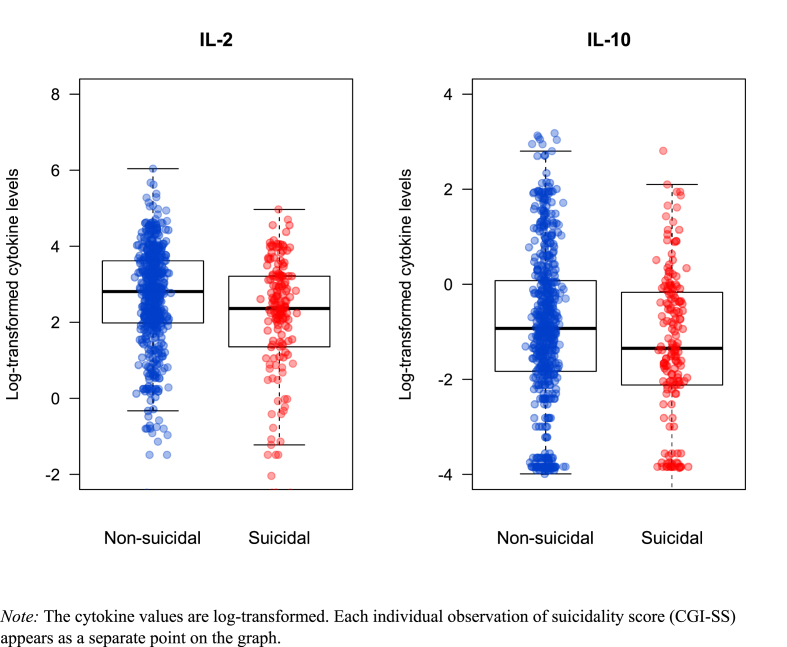
Combined box- and scatterplot showing the observed IL-2 and IL-10 values for the suicidal and non-suicidal groups. *Note:* The cytokine values are log-transformed. Each individual observation of suicidality score (CGI-SS) appears as a separate point on the graph.

**Fig. 4:**
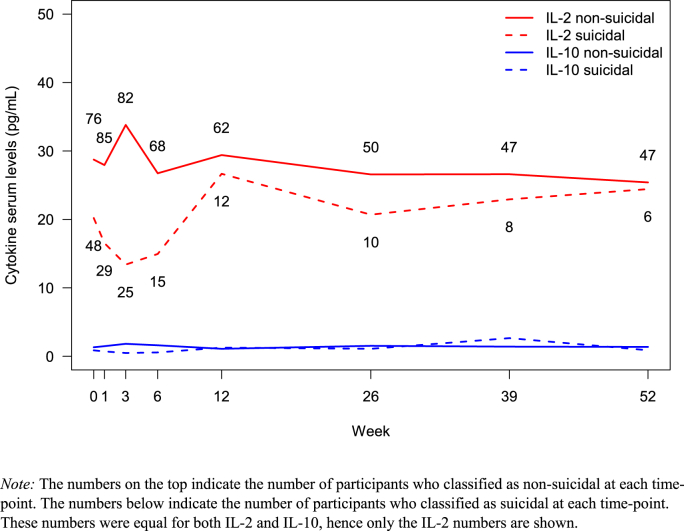
Serum levels of IL-2 and IL-10 for suicidal and non-suicidal participants during follow-up at 12 months. *Note:* The numbers on the top indicate the number of participants who classified as non-suicidal at each time-point. The numbers below indicate the number of participants who classified as suicidal at each time-point. These numbers were equal for both IL-2 and IL-10, hence only the IL-2 numbers are shown.

For the pro-inflammatory IL-2 and the anti-inflammatory IL-10, we observed different serum levels when comparing non-suicidal versus suicidal individuals, as visualized in Fig. 3. This difference was also prominent longitudinally, as shown in Fig. 4.

Table 2 shows the effect of the log-transformed cytokine values on suicidality estimated in the logistic regression model. Both our uni- and multi-variate analyses showed an inverse correlation between IL-2 and IL-10 serum levels and suicidality as measured by the CGI-SS. No other cytokines exhibited significant effects on suicidality in our sample. The sensitivity analysis using merely baseline observations showed similar results for IL-2 and IL-10, compared to the main model (data not shown).

**Table 2:** Estimated effects of cytokines on suicidality from the logistic regression model.^c^.

Cytokine	Univariate model^a^		Multivariate model^b^	
	Effect	P-value	Effect	P-value
IL-2	-0.31	0.012	-0.38	0.041
IL-4	-0.20	0.106	-0.05	0.846
IL-6	-0.08	0.573	0.14	0.433
IL-10	-0.32	0.010	-0.31	0.036
IFN-γ	-0.16	0.210	0.12	0.613
TNF-α	-0.18	0.121	0.11	0.681

*Note:*^c^The effects of cytokines are the log-effects since the cytokines in the model were log-transformed.

a Univariate model: cytokines individually tested in the model.

b Multivariate model: all six cytokines included in the model.

Results from a sensitivity analysis where CDSS item 8, suicidality, replaced CGI-SS showed consistency with the primary analysis (data not shown). The inverse cytokine-suicidality correlations were also consistent when adjusted for substance use, BMI, and CRP. In addition, we found that the type of antipsychotic medication (AP; amisulpride, aripiprazole, or olanzapine) showed no effect on suicidality.

In the sensitivity analysis including smoking as a covariate, the finding of inverse correlation between suicidality and IL-2 showed stronger significance (p-value = 0.024), whereas the IL-10 finding attenuated (p-value = 0.161).

A total of 29 participants (21.2%) had a history of substance misuse or dependence, mainly the use of cannabis (see Table 1). There was no significant difference in the cytokine levels when comparing this group with those who did not report substance use or dependence (data not shown). Furthermore, there was no significant difference in the frequency of substance use and dependence when the suicidality group and the non-suicidality group were compared at baseline (see Table 1). There was no significant difference in the frequency of cannabis use and dependence between the suicidality and the non-suicidality groups at baseline. The use of illicit substances other than cannabis was rare, resulting in too few subjects for relevant analysis. In addition, a minority of participants reported the use of illicit substances in the absence of cannabis use (three participants), in line with analyses of urine samples from the cohort (Skrede, 2021). Hence, the impact of illicit drugs other than cannabis could not be examined in the present research.

## 4 Discussion

The purpose of this study was to investigate associations between immunological factors and suicidality in a well-characterized patient cohort diagnosed with schizophrenia spectrum disorder followed with repeated measurements for 1 year. This is of special interest as patients with psychotic disorder and suicidal ideation and behaviour do not necessarily show depressive symptoms, spurring the search for independent factors constituting a potential link between inflammation and suicidality, which might ultimately serve as biomarkers.

The most prominent result of this study was that IL-2 and IL-10 serum concentrations were inversely correlated with suicidality. The inverse correlation was consistent after controlling for baseline depression, as well as CRP and substance use. This finding may be of clinical importance, as patients with active-phase psychosis may be difficult to assess clinically with regards to suicidal risk (Fredriksen et al., 2017; Fredriksen et al., 2020; Fredriksen et al., 2022; Radomsky et al., 1999). We need more knowledge about why suicidality arises and develops in schizophrenia spectrum disorder. Our sample was deemed able to provide informed consent and collaborated with the study assessments. Accordingly, we consider the associations found between the cytokine levels and suicidality to be valid.

The results concerning IL-2 cohere with previous findings (Gananca et al., 2016). An inverse correlation between suicidality and IL-2 in post-partum women with suicidal behaviour was recently shown (Brundin et al., 2021). Janeluidze et al. investigated cytokine levels in patients with major depressive disorder to distinguish between suicidal and non-suicidal patients and found a decreased IL-2 level in suicide attempters compared to non-suicidal, depressed patients (Janelidze et al., 2011).

Moreover, psychiatric side effects of immunotherapy could shed light on relevant effects of cytokines in the psychopathology of psychiatric disorders (Capuron et al., 2003). Various cytokines are approved for treatment of metastatic cancer, such as IL-2 for the treatment of melanoma and renal carcinoma (Quarantini et al., 2007; Nguyen et al., 2019). In a case report from Baron et al., a patient received IL-2 as part of melanoma treatment and shortly afterwards committed suicide (Baron et al., 1993). The administration of IL-2 is in this setting an abrupt and higher dosage than the variations in low-grade inflammation seen in psychiatric disorders. Capuron et al. investigated depressive symptoms in patients with no history of mood disorder treated with IL-2 for metastatic cancer and found a positive correlation with depressive symptoms between baseline, when IL-2 based therapy was administered, and day 5 after administration (Capuron et al., 2001). This result is not per se contradictive to our findings as we primarily investigated the association between cytokines and suicidality and not between cytokines and depression. As mentioned above depression may contribute to suicidality (Upthegrove et al., 2017; Hawton et al., 2013) but suicidal ideation can also be present independently of depression (Stanley et al., 2018; Black and Miller, 2015; Kjelby et al., 2015; Harkavy et al., 2003). The same study also found that the severity of depressive symptoms that IL-2 receiving patients exhibited was positively correlated with a concomitant increase of IL-10. Combined, these results support both our findings and display why we need further clinical studies where suicidality is assessed separately from depressive symptoms.

The second significant association in our sample was the inverse correlation between suicidality and the anti-inflammatory IL-10. Lower levels of IL-10 have been shown in depressed patients who died by suicide (Pandey et al., 2018), but suicidal behaviour in patients with schizophrenia spectrum disorder may not be directly comparable to suicidal behaviour in depression per se. In mice, Sugino et al. (2008) found an increase of IL-10 in mice treated with APs. In this context, an anti-aggressive effect of APs could play a role. Whether IL-10 plays an active or protective role should be further investigated in patients.

Das et al. found a negative correlation between IL-10 and aggression in patients with psychosis (Das et al., 2016), adding to the beforementioned thesis that suicidality in psychotic disorder may have a distinct psychopathology. Additionally, the phase of psychotic disorder should be considered. Besides the suicidality triggered by productive psychotic symptoms, post-psychotic depression is a well-known risk factor for suicidality (Guerrero-Jimenez et al., 2022). In sum, the results concerning IL-10 are complex and need further investigation.

The patients in our sample were treated with AP medication during the follow-up period. It should be considered that APs may influence cytokine levels in patients. Stapel et al. (2018) found an in vitro downregulation of IL-2 by olanzapine and aripiprazole; also in a clinical setting, a decrease of IL-2 in patients treated with APs has been showed (Miller et al., 2011; Capuzzi et al., 2017). Correlation with suicidality was not examined; it can be speculated whether a connection between a decrease of IL-2 in AP treated patients and post-psychotic depression, known to be associated with suicidality, could be relevant. Conversely, a recent review conducted by Marcinowicz et al. (2021) found that IL-2 was unaffected by AP medication, and in a review by Momtazmanesh, IL-2 was one of the non-altered cytokines in patients with schizophrenia (Momtazmanesh et al., 2019). In our sample, the type of AP showed no effect on suicidality.

As discussed earlier, former findings show that the administration of certain cytokines can lead to an increase of suicidality per se, independently of pre-existing depressive mood, shedding light on a potential causal link. According to those findings, it could be speculated that natural killer (NK) cells and T-cells could play a role in counteracting suicidality (Perry et al., 2021). IL-2 is a key cytokine with pro-inflammatory potential, promoting the expansion of NK cells and T-cells. NK cells and T-cells have been found to be increased in patients with depressive disorder (Suzuki et al., 2017). For schizophrenia, Corsi-Zuelli and Deakin also developed the contrary hypothesis and found that an initial state of excessive inflammation was followed by disturbances of dopamine and GABA function and an impaired functioning of T-cells, in particular regulatory T-cells (Tregs). Tregs are crucial for balance-keeping in ongoing inflammatory processes, and their development depends on IL-2 (Liao et al., 2011). We know from the pathogenesis of autoimmune diseases that when a relative IL-2 deficiency develops, concomitant homeostatic disturbance and chronic inflammation improve with IL-2 replacement (Grasshoff et al., 2021). In theory, treatment with IL-2 could represent a potential option for patients in suicidal crisis (Suzuki et al., 2017; Orozco Valencia et al., 2020), bearing in mind that dosage and clinical setting must be considered carefully.

## 5 Conclusions

We found an inverse correlation between serum levels of the two cytokines IL-2 and IL-10 and suicidality. Identification of inflammatory pathways implicated in suicidality could pave the way for future development of diagnostic tools and, possibly, new therapeutic approaches. Ultimately, the aim of this study was to highlight possible pathways of psychopathology and identify candidate biomarkers for use in a diagnostic and prognostic manner. In the context of former findings, IL-2 holds potential as a candidate marker predicting suicidality. Our findings support further research with respect to a potential therapeutic role of treatment with IL-2 in patients with schizophrenia who suffer from acute and severe suicidality. Further research is required to both investigate the contribution of inflammation in suicidal patients with psychosis and optimize future diagnostics and treatment.

## 6 Strengths and limitations

The long follow-up period and cohort size of 141 provided us with the opportunity to link repeated assessments of clinical symptoms with immune markers in blood samples. The clinical scores were collected by trained raters, and blood samples were drawn from fasting patients in morning hours. A challenge is that due to a pragmatic study design involving patients from an everyday clinical psychiatric practice, 18 patients had the diagnosis F23/acute and transient psychotic disorder. We did, however, choose to include those patients in our suicidality versus cytokine analyses since a significant proportion of patients exhibit other diagnoses in the years proceeding a schizophrenia diagnosis (Murrie et al., 2020). Due to the risk of masking important links and associations in this exploratory setting (type II errors), we did not adjust for multiple comparisons (Rothman, 1990). This leads to vulnerability to spurious findings (type I error) (Althouse, 2016).

In addition, we chose to compare all patients exhibiting signs of suicidality (as captured by the CGI-SS) in one group and separate them from patients with no clinical signs of increased suicidality. This led to patients with suicidal ideation and behaviour being in the same group as patients with suicide attempt(s) within the prior 7 days, and it could be argued that these patients may differ in their psychopathology. However, when looking for the possible immune signature of suicidality, one has to look where suicidality is, and dichotomization produces meaningful findings that are more easily interpreted (Farrington and Loeber, 2000). A participant with any sign of increased suicidality is therefore not classified in the non-suicidal group. Adding to this challenge is the absence of an established reference area for all cytokine concentrations in blood, making important, hypothesis-generating research challenging.

## Funding

This work was supported by the Western Norway Regional Health Authority (#911679, #911820, #F-11490) and by the 10.13039/501100005416Research Council of Norway (#213727). The funding sources had no role in study design, in the collection, analysis or interpretation of data, in the writing of this article, or in the decision to submit the article for publication.

## CRediT authorship contribution statement

**Gunnhild E. Hoprekstad:** Data curation, Formal analysis, Writing – original draft, Writing – review & editing. **Silje Skrede:** Project administration, Supervision, Writing – review & editing. **Christoffer Bartz-Johannessen:** Data curation, Formal analysis, Visualization, Writing – review & editing. **Inge Joa:** Investigation, Writing – review & editing. **Solveig K. Reitan:** Conceptualization, Investigation, Project administration, Writing – review & editing. **Vidar M. Steen:** Conceptualization, Funding acquisition, Project administration, Resources, Writing – review & editing. **Anja Torsvik:** Investigation, Project administration, Writing – review & editing. **Erik Johnsen:** Conceptualization, Funding acquisition, Investigation, Project administration, Resources, Supervision, Writing – review & editing. **Rune A. Kroken:** Conceptualization, Funding acquisition, Investigation, Project administration, Supervision, Writing – review & editing. **Maria Rettenbacher:** Conceptualization, Investigation, Project administration, Writing – review & editing.

## Declaration of competing interest

None.
