# Supplementary material for: Association between cytokines and suicidality in patients with psychosis: A multicentre longitudinal analysis
Source: Brain Behav Immun Health. 2024 Mar 20;37:100756. doi: 10.1016/j.bbih.2024.100756 (PMC10973600; doi:10.1016/j.bbih.2024.100756)
Supplement: Multimedia component 2 [file mmc2.docx]

Supplementary Figure 2: Average cytokine values and intervals containing 95% of the observations for each cytokine.
