# Supplementary material for: Association between cytokines and suicidality in patients with psychosis: A multicentre longitudinal analysis
Source: Brain Behav Immun Health. 2024 Mar 20;37:100756. doi: 10.1016/j.bbih.2024.100756 (PMC10973600; doi:10.1016/j.bbih.2024.100756)
Supplement: Multimedia component 3 [file mmc3.docx]

**Supplementary Table 1:** Distribution of CGI-SS scores at each study visit.

| Visit/CGI-SS | 1 | 2 | 3 | 4 | 5 | missing |
| --- | --- | --- | --- | --- | --- | --- |
| 1 | 76 | 35 | 9 | 1 | 3 | 7 |
| 2 | 85 | 19 | 6 | 3 | 1 | 3 |
| 3 | 82 | 20 | 5 | 0 | 0 | 6 |
| 4 | 68 | 13 | 1 | 0 | 1 | 4 |
| 5 | 62 | 10 | 1 | 1 | 0 | 4 |
| 6 | 50 | 6 | 3 | 0 | 1 | 1 |
| 7 | 47 | 4 | 3 | 1 | 0 | 0 |
| 8 | 47 | 4 | 1 | 1 | 0 | 1 |

Note: Study participants were assessed at eight study visits over a 12-month period: baseline followed by week 1, 3, 6, 12, 26, 39 and 52.
