# Supplementary material for: Association between cytokines and suicidality in patients with psychosis: A multicentre longitudinal analysis
Source: Brain Behav Immun Health. 2024 Mar 20;37:100756. doi: 10.1016/j.bbih.2024.100756 (PMC10973600; doi:10.1016/j.bbih.2024.100756)
Supplement: Supplementary file 3 [file mmc1.docx]

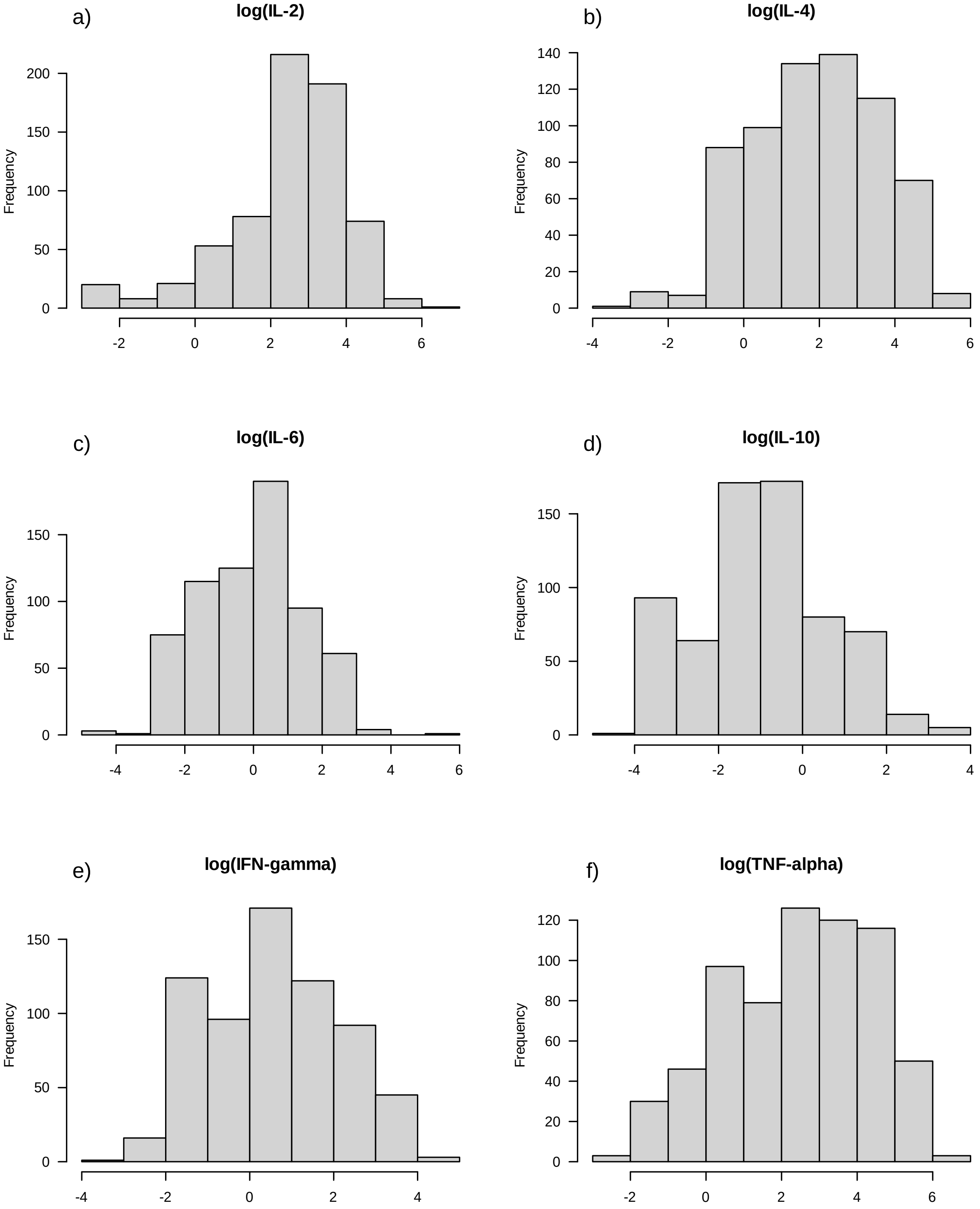

a)
log(IL-2)
Frequency
0
50
100
150
200
-2
0
2
4
6
b)
log(IL-4)
Frequency
0
20
40
60
80
100
120
140
-4
-2
0
2
4
6
c)
log(IL-6)
Frequency
0
50
100
150
-4
-2
0
2
4
6
d)
log(IL-10)
Frequency
0
50
100
150
-4
-2
0
2
4
e)
log(IFN-gamma)
Frequency
0
50
100
150
-4
-2
0
2
4
f)
log(TNF-alpha)
Frequency
0
20
40
60
80
100
120
-2
0
2
4
6

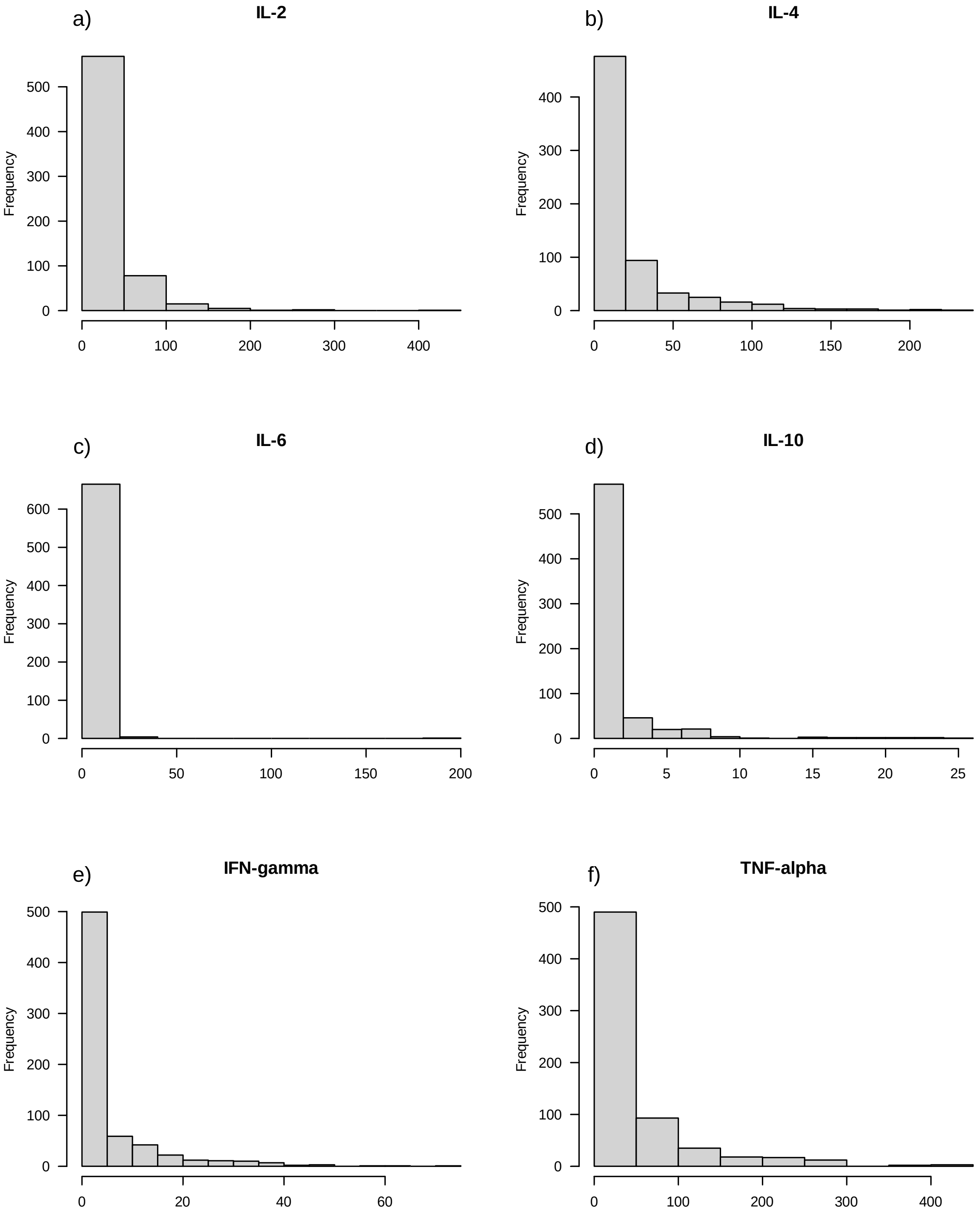

a)
IL-2
Frequency
0 100 200 300 400 500
0 100 200 300 400
b)
IL-4
Frequency
0 100 200 300 400
0 50 100 150 200
c)
IL-6
Frequency
0 100 200 300 400 500 600
0 50 100 150 200
d)
IL-10
Frequency
0 100 200 300 400 500
0 5 10 15 20 25
e)
IFN-gamma
Frequency
0 100 200 300 400 500
0 20 40 60
f)
TNF-alpha
Frequency
0 100 200 300 400 500
0 100 200 300 400
